# NeoAct: A Randomized Prospective Pilot Study on Communication Skill Training of Neonatologists

**DOI:** 10.3389/fped.2021.675742

**Published:** 2021-05-13

**Authors:** Katharina Bibl, Michael Wagner, Philipp Steinbauer, Peter Gröpel, Sabrina Wimmer, Monika Olischar, Angelika Berger, Birgit Hladschik-Kermer

**Affiliations:** ^1^Division of Neonatology, Pediatric Intensive Care and Neuropediatrics, Department of Pediatrics, Comprehensive Center for Pediatrics, Medical University of Vienna, Vienna, Austria; ^2^Division of Sport Psychology, Department of Sport Science, University of Vienna, Vienna, Austria; ^3^Department for Medical Psychology, Department for Public Health, Medical University Vienna, Vienna, Austria

**Keywords:** communication studies, communication training and development, communication training method, simulated parents, objective structural clinical examination, communication skill, neonatologists

## Abstract

**Background:** This randomized interventional study evaluated the impact of a 1-day experiential communication skills training on neonatologists' performance in doctor-parents-communication.

**Methods:** 17 neonatologists with different levels of professional experience from the Medical University of Vienna were randomized into one of two study groups: The intervention group (IG) as opposed to the control group (CG) participated in a 1-day experiential communication training. Eight weeks after the training, participants' communication skills were assessed during an objective structured clinical examination (OSCE). Neonatologists were assessed in a simulated conversation by how effectively they performed when conveying complex health-related information to parents of ill infants. Participants in the control group (CG) were assessed first during the OSCE and received their communication training later on. Self-assessment questionnaires before and after the workshop and OSCE were completed.

**Results:** The study determined that neonatologists in the IG subjectively perceived that their competence level regarding their communication skills had increased after the workshop, while this was not reflected by their performance during the OSCE assessment.

**Discussion:** A 1-day experiential communication skills training significantly increased physicians' self-evaluation concerning their communicative competence. This perceived competence did not manifest itself in increased communication skills during the OSCE.

**Conclusion:** Repeated training is needed.

## Introduction

Communication with parents of critically ill newborn or premature infants is a challenging task, even for experienced physicians. Previous studies have shown that communication skills represent an essential core skill of clinical competence for healthcare providers (1). A distinctive feature of difficult conversations in neonatology is that the primary communication is not with the patients themselves, but with the parents, who face these conversations under exceptional circumstances. Communicative skills of neonatologists have direct implications for parental satisfaction with the proposed treatment (2). These communicative skills are also associated with reduced emotional stress levels in parents in terms of fears and insecurities during the infant's hospitalization (3, 4). Poor communication skills not only impact the well-being of parents but also the health of physicians. A lack of communicative competence promotes frustration, distress, may lead to job dissatisfaction among healthcare providers and is associated with a greater risk of experiencing burnout (2).

There is substantial evidence that communication skills can be enhanced by highly structured and interactive, experiential training sessions, followed by constructive, well-balanced feedback (1). This has been shown in numerous evidence-based studies (5–7), where adequate feedback is one essential didactic element (1). Work-place based training that focuses on individual goals is considered highly effective in improving communication skills. Role plays involving standardized patients have proven to be highly realistic, as physicians are able to obtain immediate and relevant feedback about their performance through the perspective of the patient (8–10). This method allows both the training of communication skills and the promotion of empathic behavior, which leads to an increase in emotional depth in conversations between parents and physicians (11). Most previous studies on communication skills training in the field of pediatrics have predominantly focused on training sessions with actors playing the role of a sick child or adolescent (=simulated patient) (12–14). However, in the communication skills training initiative described in the current study, actors did not represent a patient, but the parents of an ill newborn infant (=simulated parents).

The aim of this pilot study was to test the implementation and immediate effectiveness of a brief (1-day) communication training session for neonatologists. Training included experiential, parent-physician scenarios involving actors. The effectiveness was evaluated 8 weeks later. We hypothesized that the proposed training session would lead to improved communication skills when compared with a control condition.

## Materials and Methods

This prospective, randomized intervention pilot study was conducted at the Department of Pediatrics and Adolescent Medicine at the Medical University of Vienna. All residents and consultants (*n* = 36) of the Division of Neonatology were invited to take part in the study, whereas 17 agreed to participate. There were no exclusion criteria as physicians were able to participate irrespective of their prior clinical experience.

### Study Procedure

**Figure 1:**
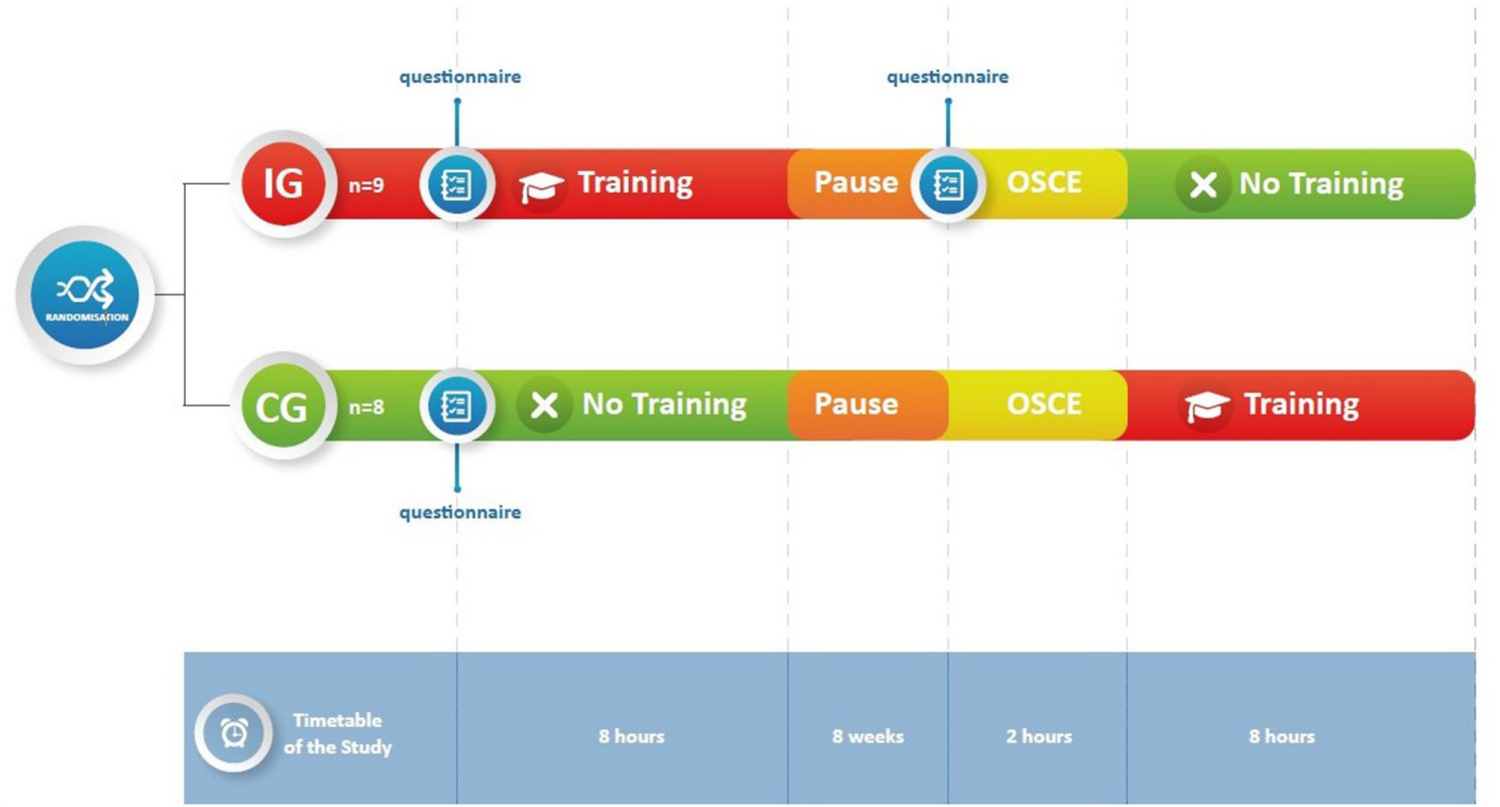
Study design.

Using a computer-generated list of random numbers (Microsoft Excel; Microsoft, Redmond, WA), we randomized participants to join either an intervention group (IG *n* = 9) or a control group (CG *n* = 8). The intervention group attended a 1-day communication training session, which included experiential scenarios and lasted for 8 h. Eight weeks later, participants completed an assessment of their communication skills. The control group completed the communication skills assessment on the same day as the intervention group, yet before attending a similar 1-day communication skills training as the IG (shown in Figure 1).

### Communication Skills Training

To minimize any interruptions during the training, all participants were released from clinical work or on-call duty for the duration of the session. All physicians signed an informed consent prior to participation and started the workshop by answering a questionnaire about their communication skills and concerns regarding communication with parents.

The course was facilitated by an experienced trainer for communication in healthcare. Training started with a brief introduction on communication theories and concepts, focusing on the specific communication needs of neonatologists. By far the most important aspect of the 1-day workshop was the experiential communication training part. Each participant blindly chose (with closed envelopes which contained the scenarios) one of two possible scenarios. The participant had to focus on the respective scenario during the communication training with standardized parents.

**Figure 2:**
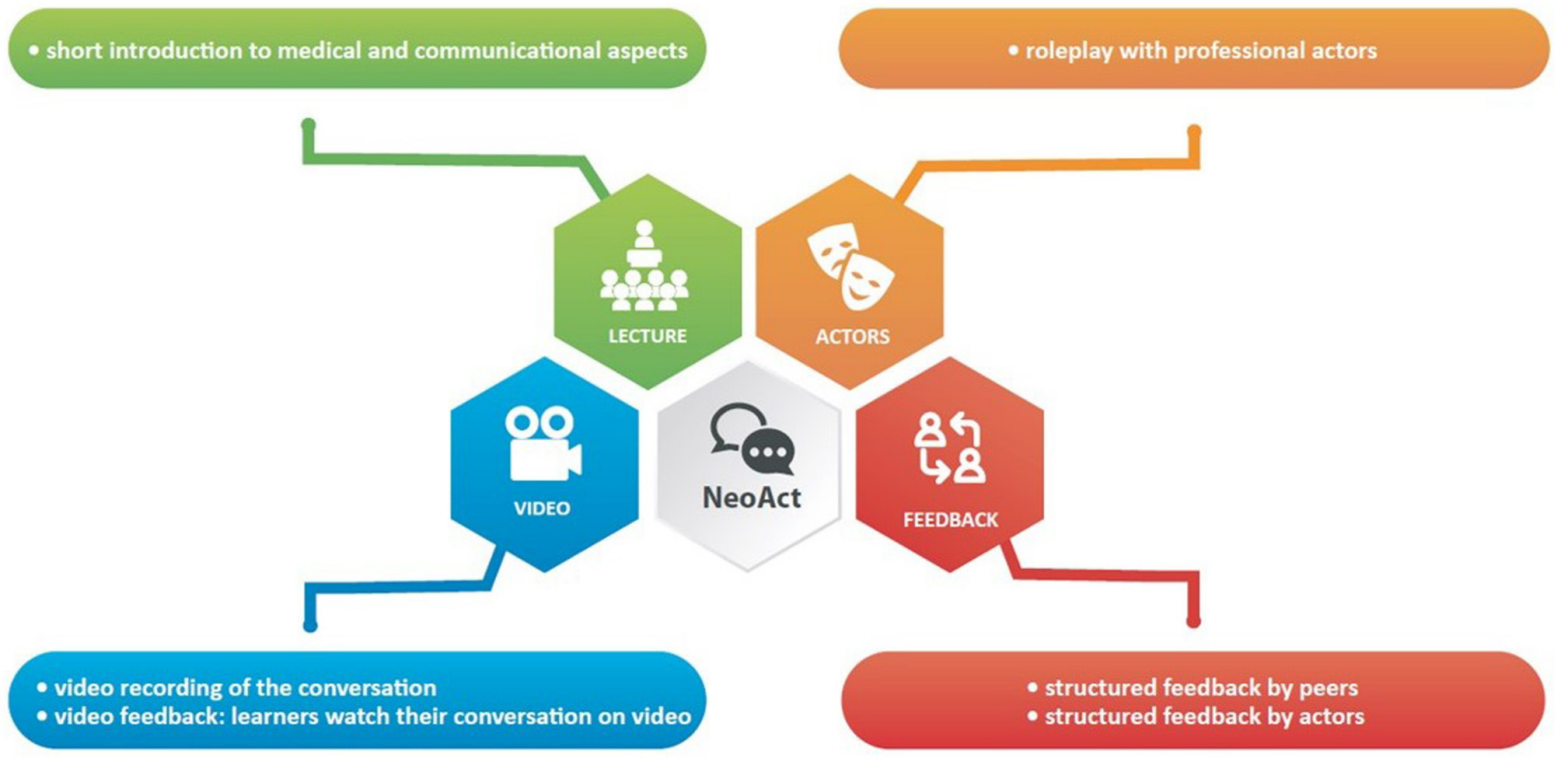
Multi-method training concept.

The trainer supported each learner in defining his or her individual learning goals for the conversation ensuing the given scenario. Learning goals included for example providing explanations that the parents can understand and remember, using an interactive approach to ensure a shared understanding of the problem or adaptive dealing with emotions Communication skills to achieve these goals were identified. Each simulated conversation was video recorded so that the participants had the option of reflecting on their performance after the conversation in a separate room. The other participants, watching the simulated conversation attentively, considered whether the trainee's pre-set goals were achieved and collected feedback. The feedback round, guided by the communication trainer, started with the participants' self-reflection. Next, the actors gave feedback from the parent's perspective, so the physician could get an insight into how his or her behavior was interpreted by the simulated parent. Finally, peers provided their feedback and suggestions for the trainee together with the expert. The training session for each participant took ~1 h. The learner was invited to do a short rehearsal, if desired. At the end, participants reflected on newly achieved skills from the training that would help them in their next conversation with parents at the Neonatal Intensive Care Unit (NICU) (shown in Figure 2).

### Scenarios

The scenarios used during the communication training session were developed by two neonatologists (KB, MW), reflecting realistic and common difficult communication situations involving parents in a NICU. The first of two possible scenarios covered the initial conversation with a father of a newborn infant with perinatal asphyxia requiring invasive ventilation and hypothermia therapy. Alternatively, in the second scenario, participants had to conduct a conversation with parents of a preterm infant born in pregnancy week 24 + 0, 9 weeks after birth and explore the social background, as the parents were not visiting their child at the NICU on a regular basis (see Annex 1). Four professional and experienced actors, who regularly work in the field of communication skills training with students at the Medical University of Vienna, practiced the scenarios under the supervision of an acting coach and neonatologists, so that the conversation during role-play would be as authentic as possible. Roles were created by the neonatologists of the study group according to their clinical experience with parents.

### Assessment of Communication Skills

In February 2018, participants' communication skills were assessed during an objective structured clinical examination (OSCE) 8 weeks after the 1-day communication training day of the intervention group. The scenario presented during the OSCE was aligned with the tasks and learning goals practiced during the training session. This allowed the assessors to rate the desired learning outcomes. Participants demonstrated their communication skills during a 5-min assessment task (task sheet see Annex 2) and were rated by two external raters who were blinded on the participants' group. Every participant performed one scenario, where he or she had to inform the mother of a premature born infant born in pregnancy week 24 + 0 about the relevance and implications of an aggravated intraventricular hemorrhage, which had been diagnosed the day before. The scenario in the OSCE was identical for all participants (see Annex 2). The raters used a standardized checklist and global rating scale to assess participants' communication skills (Annex 3). Both raters were well-trained and highly experienced in the assessment of communicative competencies.

In addition to the OSCE, participants completed a questionnaire on how they perceived their level of competence in communication, with focus on their self-confidence in and the ability to respond to the parents' needs (Annex 4). The questionnaire consisted of 24 items that were scored on a scale from strongly disagree (=1) to strongly agree (=4). A total score was computed by averaging responses to all items. Higher scores represented better communication skills. The intervention group completed the questionnaire twice, before the training and before the OSCE, whereas the control group completed the questionnaire only once, which took place on the same day the intervention group completed the post-training questionnaire (shown in Figure 1).

### Statistics

A chi-square test was computed to test whether the intervention and control groups differed in the proportion of gender, age, years at the department and position. Two-way random Intra-Class-Correlations (ICC) for absolute agreement were calculated to test the agreement between the two raters on the communication performance. Cronbach's alpha was calculated to test the internal consistency of the communication skills questionnaire. Separate *t*-tests were computed to test whether the intervention and control groups differed in their OSCE and questionnaire scores. All statistical analyses were performed using SPSS 24.0 (IBM Corp.; Armonk, NY). The level of significance was set at *p* < 0.05 (two-tailed).

### Additional Analyses

The 2 × 2 ANOVA revealed no interaction effect of group and participants' position on the OSCE communication score (*F* = 2.54, *p* = 0.14). Similarly, there was no interaction effect on self-reported communication skills (*F* = 0.66, *p* = 0.43).

## Results

### Participants Characteristics

Between December 2017 and March 2018, 17 physicians completed their communication training and assessment of their communication skills. In the intervention group (*n* = 9), five participants were residents and four were consultants. The control group (*n* = 8) consisted of five residents and three consultants. The groups did not differ in gender, age, years at the department, and position (*p* > 0.55). Participants' characteristics are shown in Table 1.

**Table 1:** Participants characteristics.

**Characteristics**	**Intervention group (*n* = 9)**	**Control group (*n* = 8)**	**Chi-square**	***p*-value**
**Gender**			0.03	0.86
Male	3 (33)	3 (38)		
Female	6 (67)	5 (62)		
**Age**			2.12	0.55
20–30 years	3 (33)	5 (63)		
30–40 years	1 (11)	1 (12)		
40–50 years	4 (44)	2 (25)		
50–60 years	0 (0)	0 (0)		
60–70 years	1 (11)	0 (0)		
**Employed at the department for:**			0.59	0.75
0–3 years	4 (44)	5 (63)		
4–10 years	2 (22)	1 (12)		
>10 years	3 (33)	2 (25)		
**Position**			0.08	0.77
Resident	5 (56)	5 (63)		
Consultant	4 (44)	3 (37)		

### Expert-rated Communication Skills (OSCE-Scores)

The inter-rater agreement between two communication raters was 0.75. The intervention and control groups did not differ in the OSCE communication score (*t* = 0.79, *p* = 0.45). Means and standard deviations of the OSCE score for the two groups are presented in Table 2.

**Table 2:** Communication scores of the two study groups.

**Communication score**	**Intervention group (*n* = 9)**	**Control group (*n* = 8)**	***t*-test**	***p*-value**
Expert-rated (OSCE)	16.33 ± 3.51	17.56 ± 2.86	0.79	0.445
Self-reported	3.09 ± 0.37	2.73 ± 0.27	2.26	0.039

*Parameters are shown as mean ± SD. The expert-rated (OSCE) score can range from 0 to 26 and the self-reported score can range from 1 to 4, with higher scores representing better communication skills. OSCE, objective structured clinical examination*.

### Self-reported Communication Skills

The communication questionnaire showed a high internal consistency (α = 0.92). Participants in the intervention group reported better communication skills after their training (M = 3.07, SD = 0.37) than before (M = 2.95, SD = 0.37), but this improvement was only marginally significant (*t* = 1.95, *p* = 0.087). However, there was a significant difference between the intervention group's self- reported post-training communication skills and that of the control group (*t* = 2.26, *p* = 0.039), with the intervention group reporting a higher ability to communicate with parents (Table 2).

## Discussion

This randomized prospective intervention pilot study investigated the effect of a 1-day communication training initiative for neonatologists on their communication skills during a difficult conversation with simulated parents (actors) of a sick newborn child. To our knowledge, this is the first study of its kind in the field of neonatology.

As mentioned previously, communication skills represent an essential skill of clinical competence for healthcare providers in all clinical fields (1). Therefore, communication skills training with simulated patients has become of relevance in the field of medical education (9, 15). However, residents admit to be far less prepared for difficult conversations with pediatric patients and their relatives than they are for adult patients (16). In the field of pediatrics, several curricular and communication techniques have been implemented in different hospitals to improve pediatricians' communication skills. As a consequence, this has led to higher parent satisfaction and confidence of physicians in their communication skills (17, 18). Still, in the field of neonatology, there is a distinct need for better clinician-parent communication during neonatal hospitalization (19).

In our study, we could not find significant differences between the intervention and the control group concerning expert-rated communication skills in the OSCE. This does not support recent evidence that communication skills training usually shows remarkable improvement in these skills (20–22). Even after having run interaction analysis with physicians' clinical experience level being the moderator variable, no differences were found. This contradicts known psychological phenomena, which show that behavioral change is achieved more easily the younger participants are (23). It is well-known, that certain patterns physicians acquire during their clinical practice are a challenge to change in retrospect (24). However, this study could not confirm these phenomena. This may be explained by the study design, applying only a 1-day communication skills training session. It is evident that for skills to be retained long-term, repeated training sessions are necessary. It has been suggested that attending at least three training sessions with several weeks' break in between is effective for improving communication skills sustainably (1, 25).

With reference to self-reported communication skills, participants in the intervention group reported a higher ability to communicate with parents before the OSCE compared to the control group. They presumably felt more comfortable and confident in such situations. This finding is in line with numerous previous studies in pediatric and adult medicine proving the beneficial effect of communication skills training on self-confidence and comfort level of physicians during difficult conversations (15, 26, 27). In 2015, Lechner et al. showed that neonatologists who attended a workshop focusing on managing difficult conversations during their fellowship displayed high levels of satisfaction when leading difficult conversations with parents of a sick newborn (20).

A meaningful strength of our study was the multi-method approach. The training sessions included several teaching and learning approaches, such as short didactics, communication skills training with simulated parents and feedback by actors (simulated parents) as well as peers. This combination of different learning strategies with a focus on simulation and structured feedback has been highly recommended in guidelines developed for achieving improvements in communication skills (17, 20). Practice orientated training sessions including peer-simulation and scenarios with simulated patients play a key role in their efficiency and are thus optimal educational methods for adults (15). Recently, it has been shown that the use of formally trained family members of former patients as simulated parents is very effective in improving core competencies in communication of neonatal fellows (28).

This study is the first randomized controlled study on the impact of targeted training in communication skills of neonatologists, with a standardized assessment in a simulated scenario of both the intervention and the control group. No other study in the field of neonatology has combined subjective self-assessment of participants and objective skills assessment by experts to rate neonatologists' communicative competence. This pilot study found that a 1-day training session may improve physicians self-confidence in communicating with parents according to the self-reported communication questionnaires. However, there is no evidence of increased communicative performance, irrespective of the physician's experience.

### Limitations

The design of this study implies few notable limitations. First of all, the overall sample size of 17 participants was small. However, both groups represented a satisfactory range of experience levels of neonatologists and are, therefore, a realistic representation of clinical staff of a NICU.

Although participants rated the training concept as highly efficient during debriefing, they would have preferred more than two scenarios for the whole group. In fact, a wider variety of scenarios clearly would have diversified training. Nevertheless, the repeated practice of those scenarios by different participants allowed for discussion about a variety of approaches to a specific situation.

The physicians' increase in self-confidence in communicating with parents was measured by using self-reported communication questionnaires and thus just reflects physicians' self-perceived confidence levels. Therefore, these results have to be interpreted with caution of course.

This pilot study has been conceived with a view to conducting more research into the effectiveness of ongoing training in communication and interpersonal skills for neonatologists in a randomized setting.

## Data Availability Statement

The raw data supporting the conclusions of this article will be made available by the authors, without undue reservation.

## Ethics Statement

For this study ethics approval was not required according to the ethics committee of the Medical University of Vienna. The study as well as the questionnaires were approved by the data protection committee of the Medical University of Vienna. All participant signed a written informed consent prior to participation in the study.

## Author Contributions

KB, MW, and BH-K substantially contributed to conception and design, acquisition of data, drafting the article and revising it critically for important intellectual content and final approval of the version to be published. PS and PG substantially contributed to analysis and interpretation of data and revising the manuscript critically for important intellectual content. SW substantially contributed to acquisition of data and revising the article critically for important intellectual content. MO and AB substantially contributed to revising the article critically for important intellectual content and final approval of the version to be published. All authors approved the final manuscript as submitted and agreed to be accountable for all aspects of the work.

## Conflict of Interest

The authors declare that the research was conducted in the absence of any commercial or financial relationships that could be construed as a potential conflict of interest. The reviewer HL-S declared a shared affiliation, with no collaboration, with the authors KB, MW, PS, PG, SW, MO, AB, and BH-K to the handling editor at the time of the review.
