# Supplementary material for: NeoAct: A Randomized Prospective Pilot Study on Communication Skill Training of Neonatologists
Source: Front Pediatr. 2021 May 13;9:675742. doi: 10.3389/fped.2021.675742 (PMC8158577; doi:10.3389/fped.2021.675742)
Supplement: Supplementary file 1 [file Data_Sheet_1.docx]

**Annex 1. Standardized scenarios for communication skills training**

Each participant had to perform one of the two following scenarios with simulated parents in the communication skills training

| **Scenario 1** | **Title** | **Admission of an asphyxiated newborn** |
| --- | --- | --- |
|  | Patient history | Gestational age 40+1, meconium aspiration syndrome, initially floppy infant without any breathing effort. The newborn was intubated and transferred to the local NICU. Hypothermia therapy was started. The father of the newborn arrives at the NICU and the physician needs to talk about the diagnosis and treatment. |
|  | Participant’s task | Inform the father about the medical state and situation of his child and further treatment options. |
|  | Situation in the scenario | The father is in a state of shock, worried, and asks the same questions repeatedly. |
|  | Learning goals | The goal is to find the right setting for the conversation and to convey information about the child’s diagnosis and further procedures to the father adjusting the information to his individual capacity to process information. |

| **Scenario 2** | **Title** | **Parent’s integration of a long-term patient** |
| --- | --- | --- |
|  | Patient history | The patient was born in gestational age 24+0 after premature rupture of the membranes two days prior to birth and signs of congenital infection. After C-section, the baby showed no breathing effort and therefore needed intubation initially and stayed on the ventilator until 30 weeks of GA. The patient also suffered from an intraventricular hemorrhage grade III on the left side of the brain. The patient is now in week 33+1 and on nasal cannula with 40% oxygen. The parents are not visiting their child on a regular basis and previous attempts of the nurses to integrate the parents in the care of their child were not successful until now. |
|  | Participant’s task | Have a conversation with both parents to explore the family’s social environment and how to integrate them further into the infant’s care. |
|  | Situation in the scenario | The parents are very anxious about the situation of having a sick preterm baby and feel overwhelmed by it (father has problems at work, another young sibling at home, etc.) |
|  | Learning goals | The goal is to find the right setting for this conversation to convey information about the need of parent integration for the child’s benefit and to figure out reasons for the parent behavior without making them feel guilty. |

**Annex 2. Exercise sheet for physicians at OSCE**

**Medical background:**

Felix is a 9-week old premature child admitted to your neonatal intensive care unit. After his delivery in pregnancy week 24+0, various complications including long-term invasive ventilation, infections and hemodynamic instability occurred. Furthermore, a previously known intraventricular hemorrhage was aggravated during the time of clinical instability (now diagnosed as IVH° IV on the right, and IVH° III on the left side of the brain). The parents were made aware of the aggravation yesterday, but have not had a detailed conversation about the implications of this diagnosis until now.

In this conversation, it is your task to inform the mother about the relevance and implications of the aggravated intraventricular hemorrhage.

A motor impairment is very likely for the future. However, the impact of the hemorrhage on mental development cannot be predicted at that moment.

Please do not include other complications due to prematurity in the conversation. The conversation should only focus on the diagnosis and implications of intraventricular hemorrhage.

**Your Task:**

1) Inform the mother about the diagnosis and relevance of the aggravated intraventricular hemorrhage as well as of the probable consequences.

2) Pay attention to the mother’s need for information and her capacity to process new information.

3) Involve the mother’s emotional reactions and react adequately. Offer specific help when needed.

Timeframe: 5 minutes

**Annex 3. The objective structured clinical examination (OSCE) of a physician’s communication skills**

| **Criterion** | **Points (max.)** |
| --- | --- |
| Physician informs the parent about the consequences of the already known cerebral hemorrhage and that future physical and mental development of the child cannot be predicted at present … |  |
| - uses understandable/adequate language (talks slowly, no medical terms, short sentences) | 2 |
| - can summarize essential information | 1 |
| - after explaining the possibility of medical impairments, the physician takes a break to allow space for the parent’s reaction | 1 |
| Physician involves the parent’s needs for further information and adapts the conversation depending on their capacity to process new information |  |
| - asks about previous knowledge | 1 |
| - asks about specific information needs | 1 |
| - encourages parents to ask questions | 1 |
| - is responsive to the parent’s needs | 1 |
| - clarifies parent’s comprehension | 1 |
| - summarizes understandibly | 1 |
| Physician recognizes emotional reactions and reacts accordingly… |  |
| - directly asks parents about their emotions | 2 |
| - clearly verbalizes preceived emotions | 1 |
| - articulates understanding for emotions | 1 |
| - offers support | 2 |
| General communication aspects… |  |
| - demonstrates active listening | 2 |
| - can convey information clearly to the parents | 2 |
| - is responsive to parent’s needs and means | 2 |
| - acts empathically and respectfully | 2 |
| - establishes a feasible physician-parent communication basis | 2 |

**Annex 4. The communication skills questionnaire**

For each item, indicate how much you agree or disagree with what the item says.

|  | strongly disagree | disagree | agree | strongly agree |
| --- | --- | --- | --- | --- |
| 1. I have adequate communication skills | ➀ | ➁ | ➂ | ➃ |
| 1. Communication with parents is an essential part of my medical activities | ➀ | ➁ | ➂ | ➃ |
| 1. I am satisfied with my communication skills with parents | ➀ | ➁ | ➂ | ➃ |
| 1. I am under the impression that I can structure discussions in terms of time properly | ➀ | ➁ | ➂ | ➃ |
| 1. I can convey information in a way that the other person understands | ➀ | ➁ | ➂ | ➃ |
| 1. It is easy for me to deal with criticism from the parents | ➀ | ➁ | ➂ | ➃ |
| 1. I feel confident when dealing with anxious parents | ➀ | ➁ | ➂ | ➃ |
| 1. I am able to establish a positive and sustainable doctor-parent relationship | ➀ | ➁ | ➂ | ➃ |
| 1. I am able to adapt a conversation to the occasion and needs for the parents | ➀ | ➁ | ➂ | ➃ |
| 1. I am able to schedule a conversation | ➀ | ➁ | ➂ | ➃ |
| 1. I am able to collect and convey information in a structured way | ➀ | ➁ | ➂ | ➃ |
| 1. I can deal adequately with emotions of the parents | ➀ | ➁ | ➂ | ➃ |
| 1. I can reflect my emotions and behavior after a conversation | ➀ | ➁ | ➂ | ➃ |
| 1. I know what to look for when delivering bad news to parents | ➀ | ➁ | ➂ | ➃ |
| 1. I know my strengths and weaknesses in relation to medical interviewing | ➀ | ➁ | ➂ | ➃ |
| 1. I have reliable information to prepare for conversations with parents | ➀ | ➁ | ➂ | ➃ |
| 1. I think I am able to fulfill the communicative requirements set for me | ➀ | ➁ | ➂ | ➃ |
| 1. I am under the impression that the parents are largely satisfied with my interviewing | ➀ | ➁ | ➂ | ➃ |
| 1. I feel confident when dealing with overwhelmed parents | ➀ | ➁ | ➂ | ➃ |
| 1. I am able to deal adequately with parents who question my professional competencies | ➀ | ➁ | ➂ | ➃ |
| 1. I am able to talk to parents about the uncertain prognosis of their child | ➀ | ➁ | ➂ | ➃ |
| 1. I can find a common consensus on how to proceed with parents whose behavior towards the child does not meet my expectations | ➀ | ➁ | ➂ | ➃ |
| 1. I make sure to get a clear picture of the situation and needs of each parent during a conversation with the parents | ➀ | ➁ | ➂ | ➃ |
| 1. I feel well prepared for conversations with parents | ➀ | ➁ | ➂ | ➃ |
