# Supplementary figures and images for: NeoAct: A Randomized Prospective Pilot Study on Communication Skill Training of Neonatologists
Source: Front Pediatr. 2021 May 13;9:675742. doi: 10.3389/fped.2021.675742 (PMC8158577; doi:10.3389/fped.2021.675742)

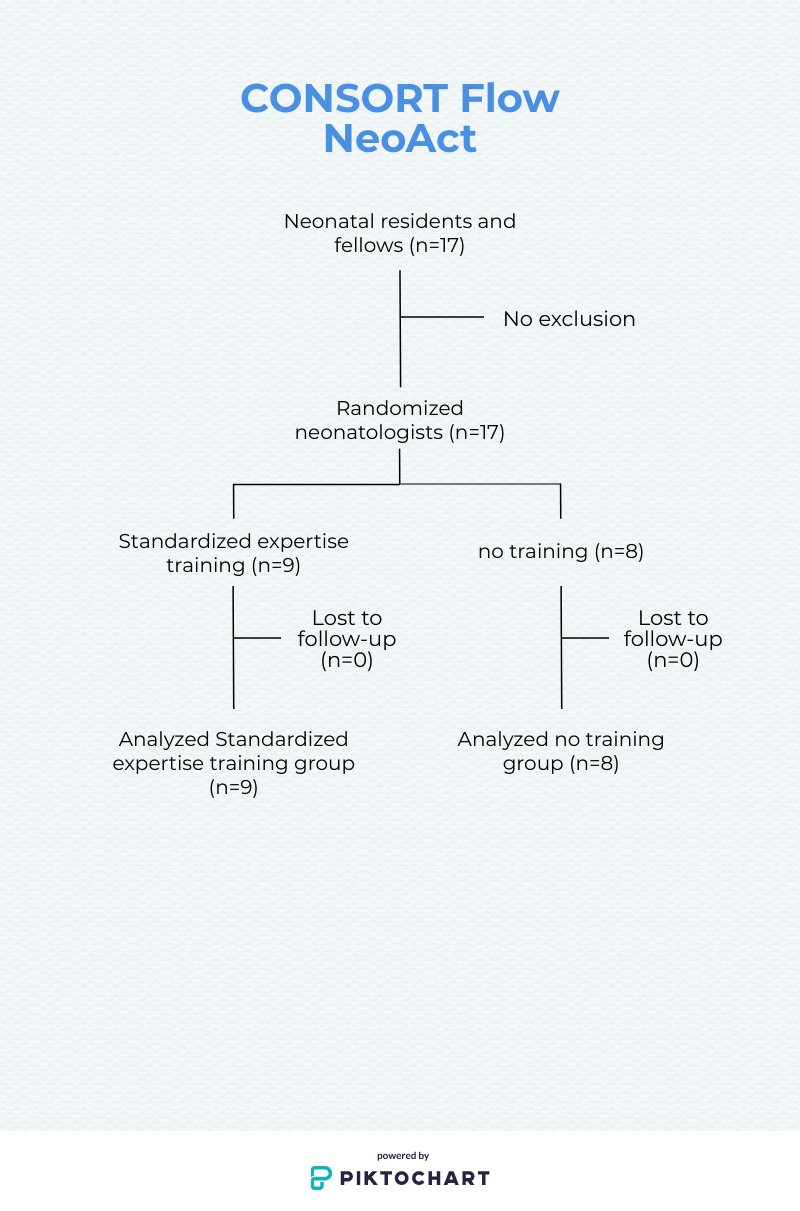

Supplement: Supplementary file 2 [file Image_1.PNG]
